# Supplementary material for: Rational engineering of minimally immunogenic nucleases for gene therapy
Source: Nat Commun. 2025 Jan 2;16:105. doi: 10.1038/s41467-024-55522-1 (PMC11696374; doi:10.1038/s41467-024-55522-1)
Supplement: Supplementary file 1 — Supplementary Information [file 41467_2024_55522_MOESM1_ESM.pdf]

Supplementary Information

Supplementary Figures 1 – 11
Supplementary Table 1
Supplementary Data 1 – 8
Supplementary Code
Source Data

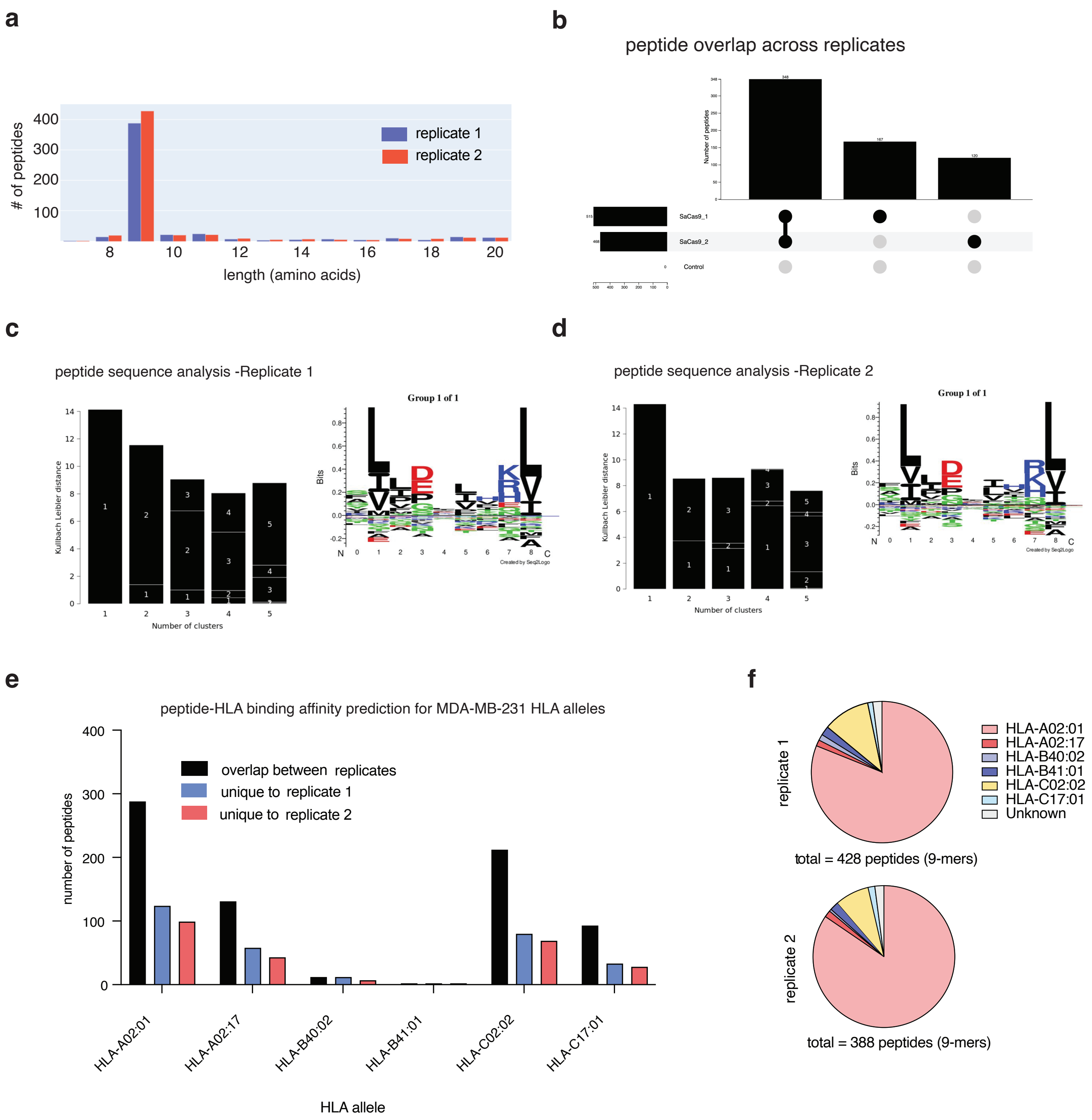

Supplementary Figure 1. Immunopeptidomics data quality analysis for SaCas9 replicates. (a) Histogram of frequency distribution of peptide lengths across replicates. **(b)** Upset plot of two SaCas9 replicates depicting peptide overlap across replicates. **(c)** GibbsCluster 2.0 (Gibbs Clustering) applied to Replicate 1 peptides after pre-processing. Binding motif analysis uses Kullbach Leibler Distance (KLD) measurement for various clusters and on the right, the logo plot of the major peptide cluster. **(d)** GibbsCluster 2.0 (Gibbs Clustering) applied to Replicate 2 peptides after pre-processing. Binding motif analysis uses Kullbach Leibler Distance (KLD) measurement for various clusters and on the right, the logo plot of the major peptide cluster. **(e)** Predicted number of binders to each HLA-allele expressed by MDA-MB0231 cells applied after pre-processing through Immunolyser analysis. **(f)** Number of binders to the six MDA-MB 231 HLA-alleles and the number of unknown binders (non-MDA-MB 231 HLA alleles) within the 9-mers of the peptide list of Replicate 1 (above) and Replicate 2 (below).

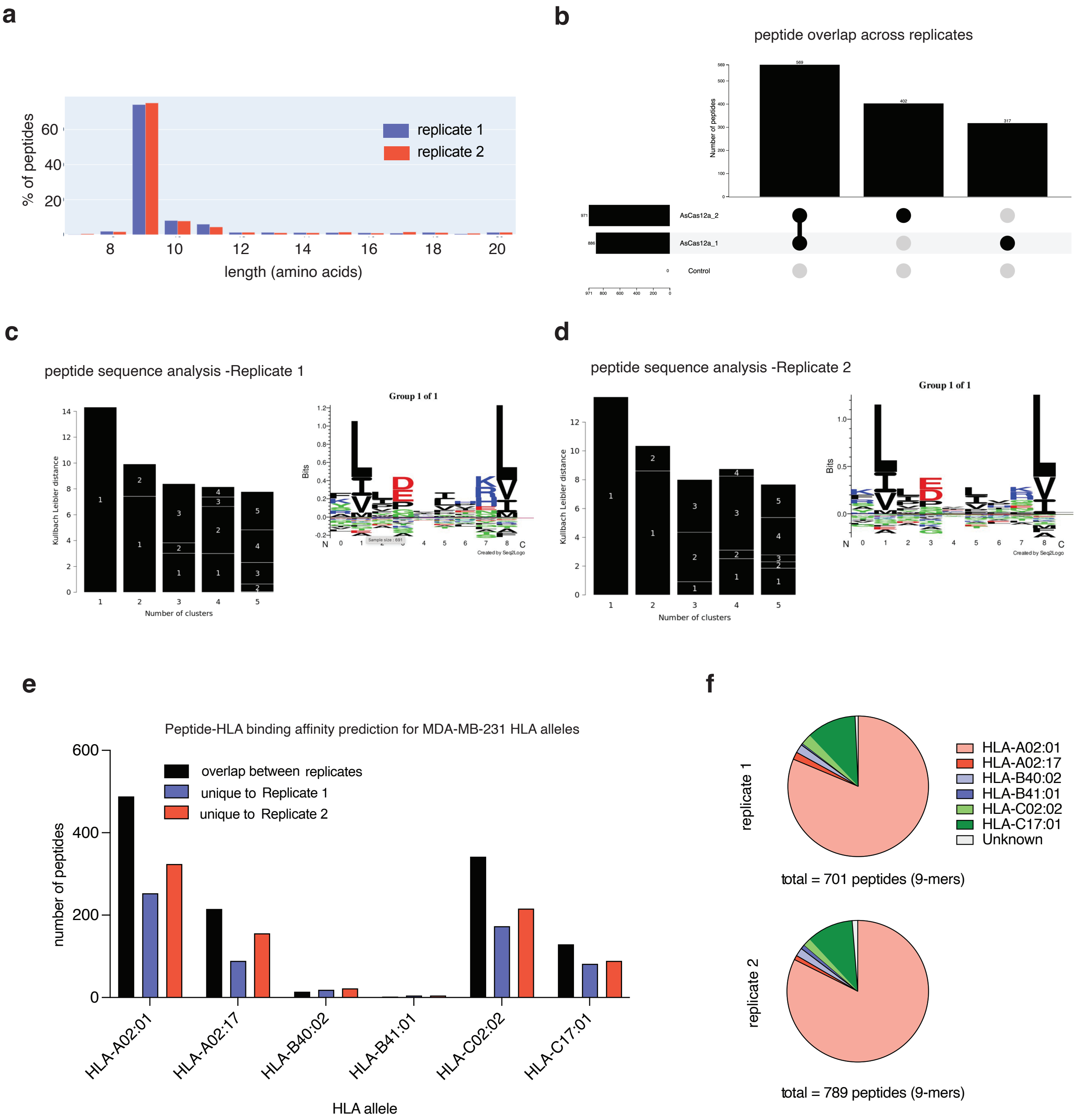

Supplementary Figure 2. Immunopeptidomics data quality analysis for AsCas12a replicates. (a) Histogram of frequency distribution of peptide lengths across replicates. **(b)** Upset plot of two AsCas12a replicates depicting peptide overlap across replicates.**(c)** GibbsCluster 2.0 (Gibbs Clustering) applied to Replicate 1 peptides after pre-processing. Binding motif analysis uses Kullbach Leibler Distance (KLD) measurement for various clusters and on the right, the logo plot of the major peptide cluster. **(d)** GibbsCluster 2.0 (Gibbs Clustering) applied to Replicate 2 peptides after pre-processing. Binding motif analysis uses Kullbach Leibler Distance (KLD) measurement for various clusters and on the right, the logo plot of the major peptide cluster. **(e)** Predicted number of binders to each HLA-allele expressed by MDA-MB0231 cells applied after pre-processing through Immunolyser analysis. **(f)** Number of binders to the six MDA-MB 231 HLA-alleles and the number of unknown binders (non-MDA-MB 231 HLA alleles) within the 9-mers of the peptide list of Replicate 1 (above) and Replicate 2 (below).

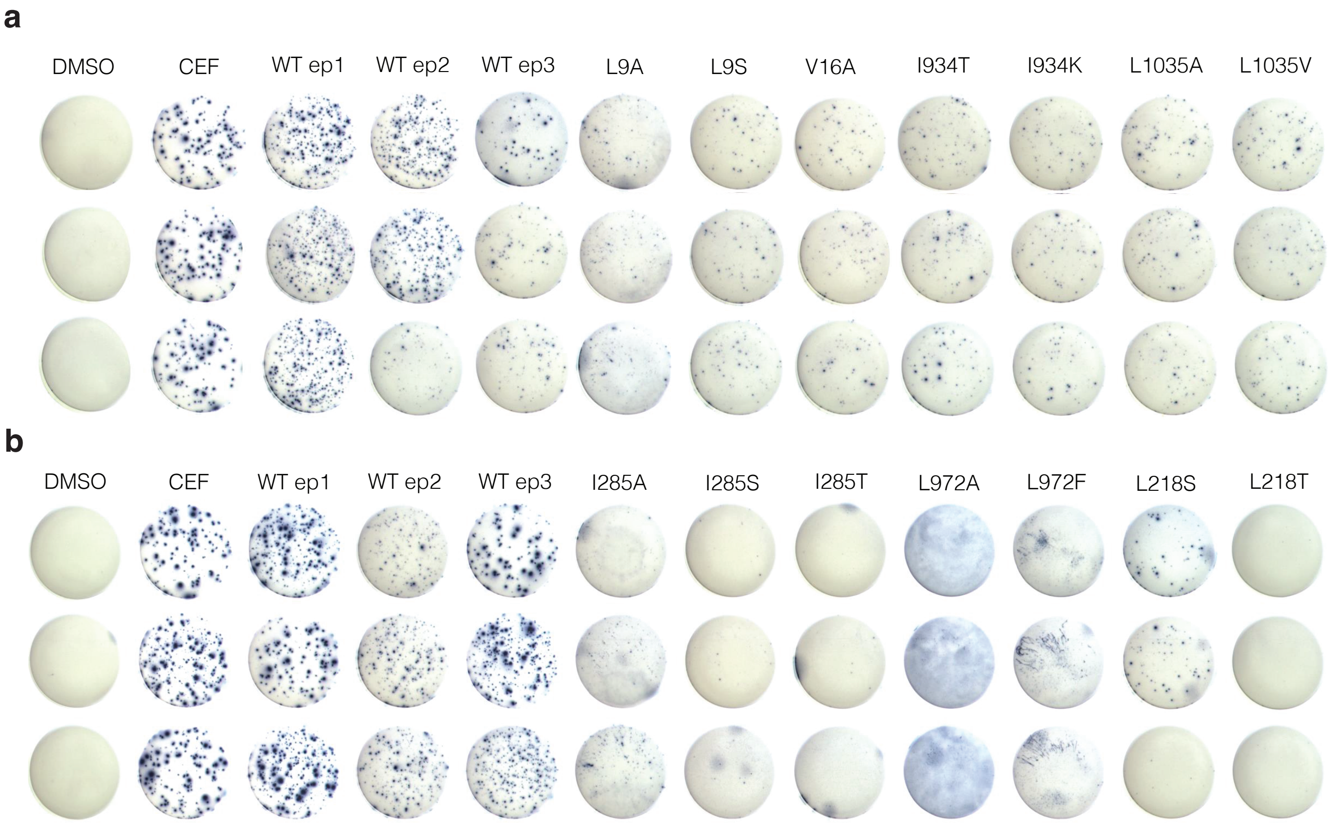

Supplementary Figure 3. Expanded ELISpot images of SaCas9 and AsCas12a peptides. (a) Full ELISpot images for patient 1 treated with SaCas9 peptides and variants. **(b)** Full ELISpot images for patient 1 treated with AsCas12a peptides and variants.

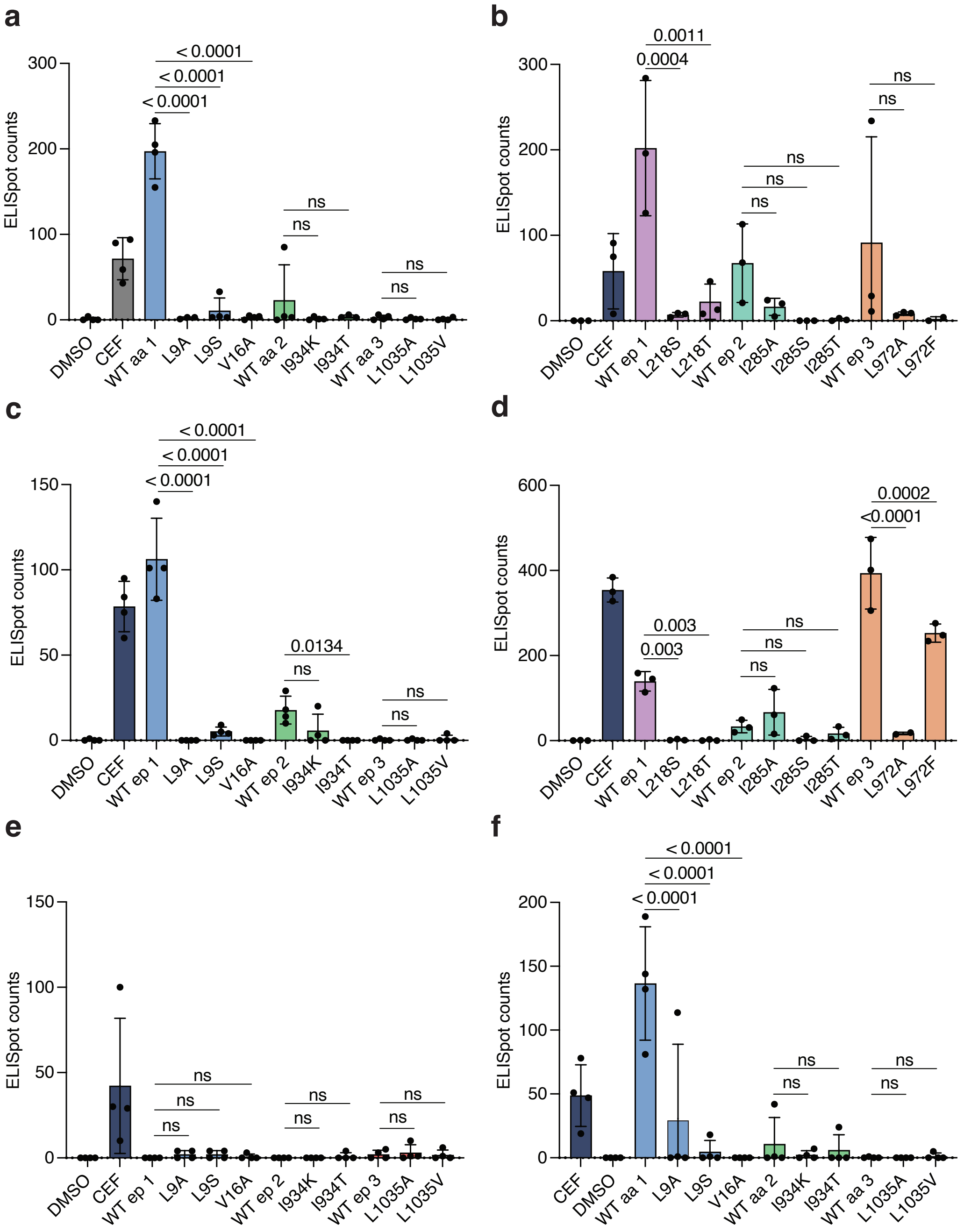

Supplementary Figure 4. ELISpot quantification with HLA-A*0201 patient samples. (a) ELISpot quantification for patient 2 treated with SaCas9 peptides and variants.**(b)** ELISpot quantification for patient 2 treated with AsCas12a peptides and variants. **(c)** ELISpot quantification for patient 3 treated with SaCas9 peptides and variants. **(d)** ELISpot quantification for patient 3 treated with AsCas12a peptides and variants. **(e)** ELISpot quantification for patient 4 treated with SaCas9 peptides and variants. **(f)** ELISpot quantification for patient 5 treated with SaCas9 peptides and variants.

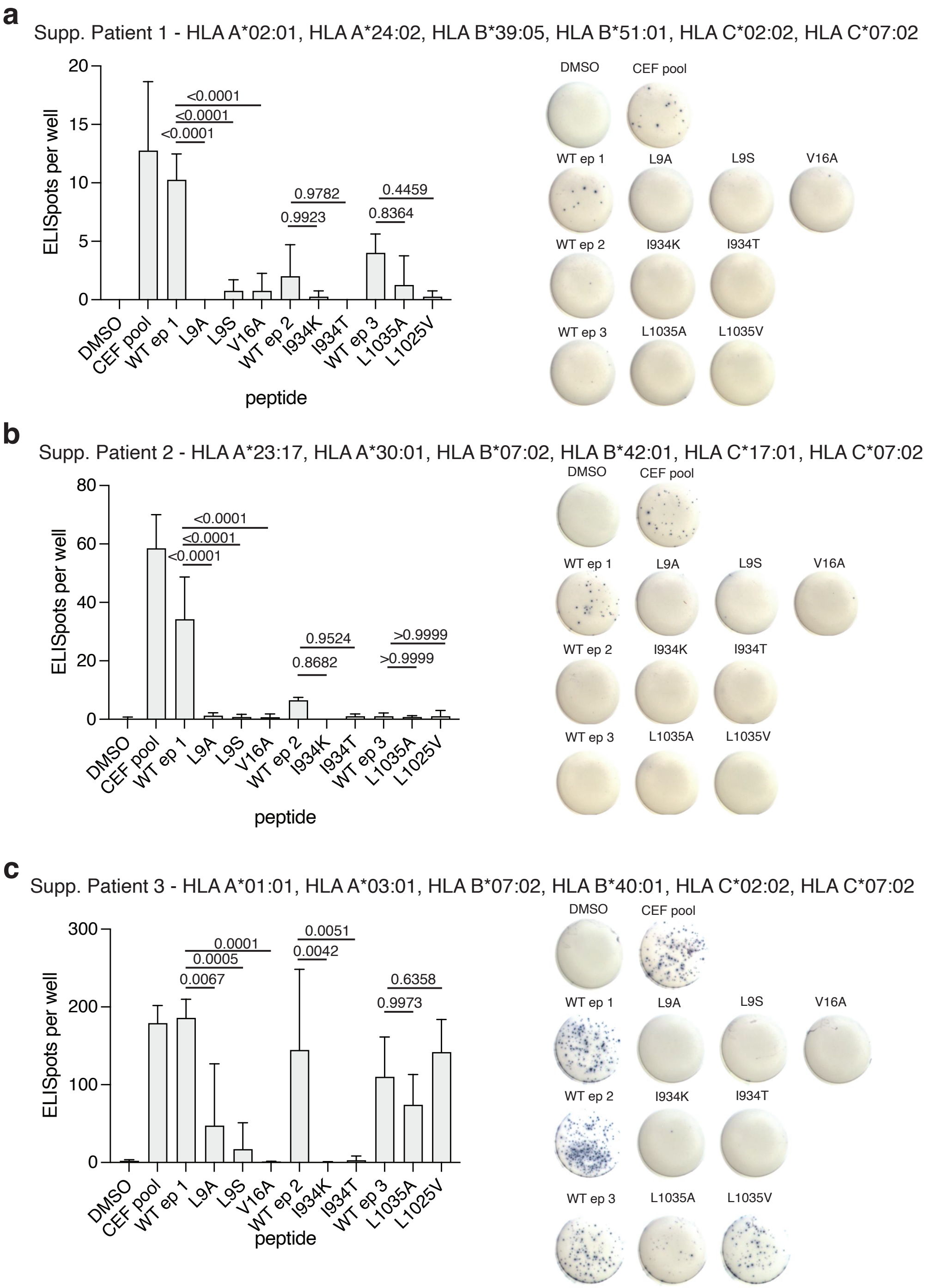

Supplementary Figure 5. ELISpot quantification with diverse HLA patient samples. (a) ELISpot quantification for HLA A*02:01, HLA A*24:02, HLA B*39:05, HLA B*51:01, HLA C*02:02, HLA C*07:02 patient treated with SaCas9 peptides and variants. **(b)** ELISpot quantification for HLA A*23:17, HLA A*30:01, HLA B*07:02, HLA B*42:01, HLA C*17:01, HLA C*07:02 patient treated with SaCas9 peptides and variants. **(c)** ELISpot quantification for HLA A*01:01, HLA A*03:01, HLA B*07:02, HLA B*40:01, HLA C*02:02, HLA C*07:02 patient treated with SaCas9 peptides and variants. Significance of comparisons was assessed with a one-way ANOVA with a p-value threshold of 0.05.

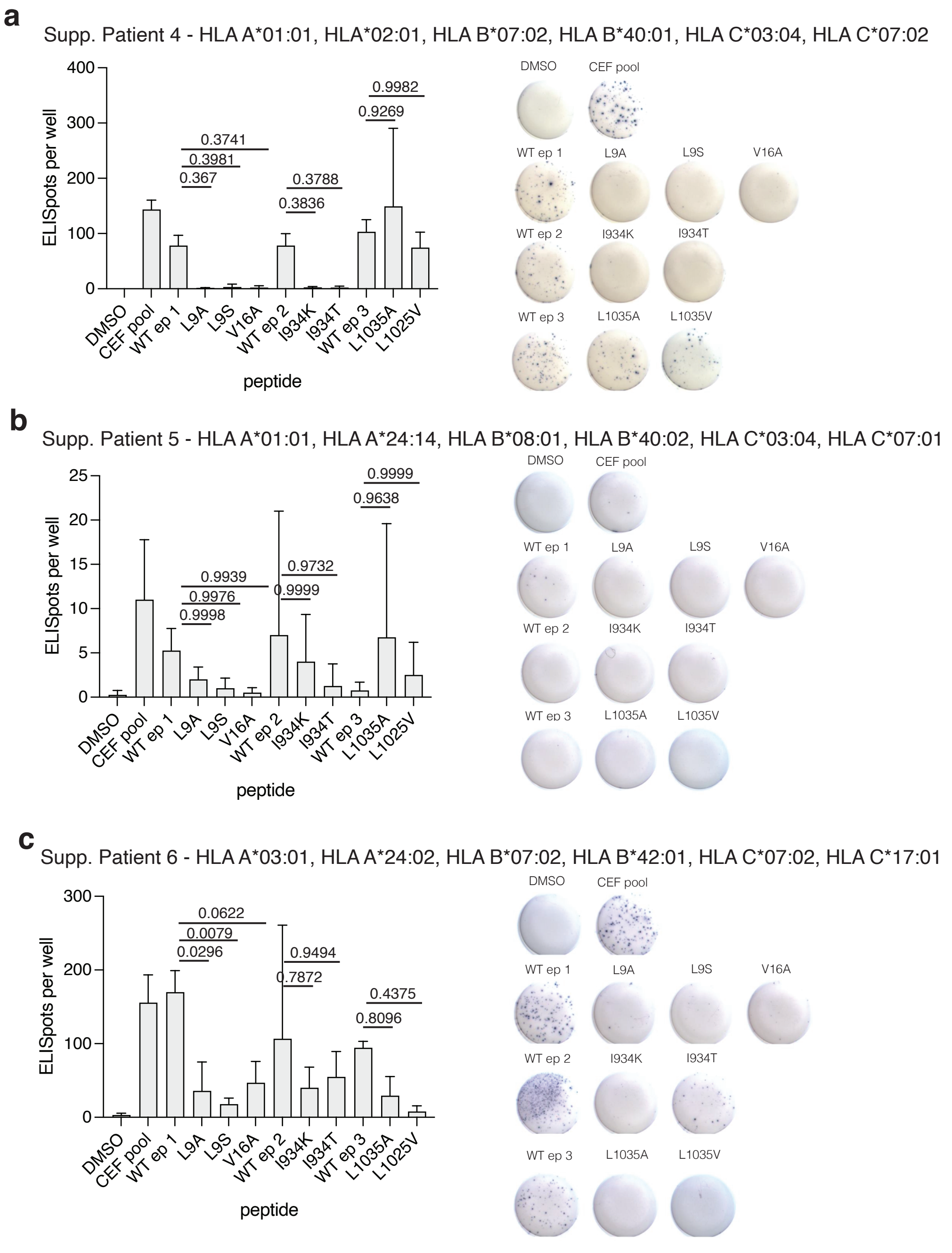

Supplementary Figure 6. ELISpot quantification with diverse HLA patient samples. (a) ELISpot quantification for HLA A*01:01, HLA*02:01, HLA B*07:02, HLA B*40:01, HLA C*03:04, HLA C*07:02 patient treated with SaCas9 peptides and variants.**(b)** ELISpot quantification for HLA A*01:01, HLA A*24:14, HLA B*08:01, HLA B*40:02, HLA C*03:04, HLA C*07:01 patient treated with SaCas9 peptides and variants.**(c)** ELISpot quantification for HLA A*03:01, HLA A*24:02, HLA B*07:02, HLA B*42:01, HLA C*07:02, HLA C*17:01 patient treated with SaCas9 peptides and variants. Significance of comparisons was assessed with a one-way ANOVA with a p-value threshold of 0.05.

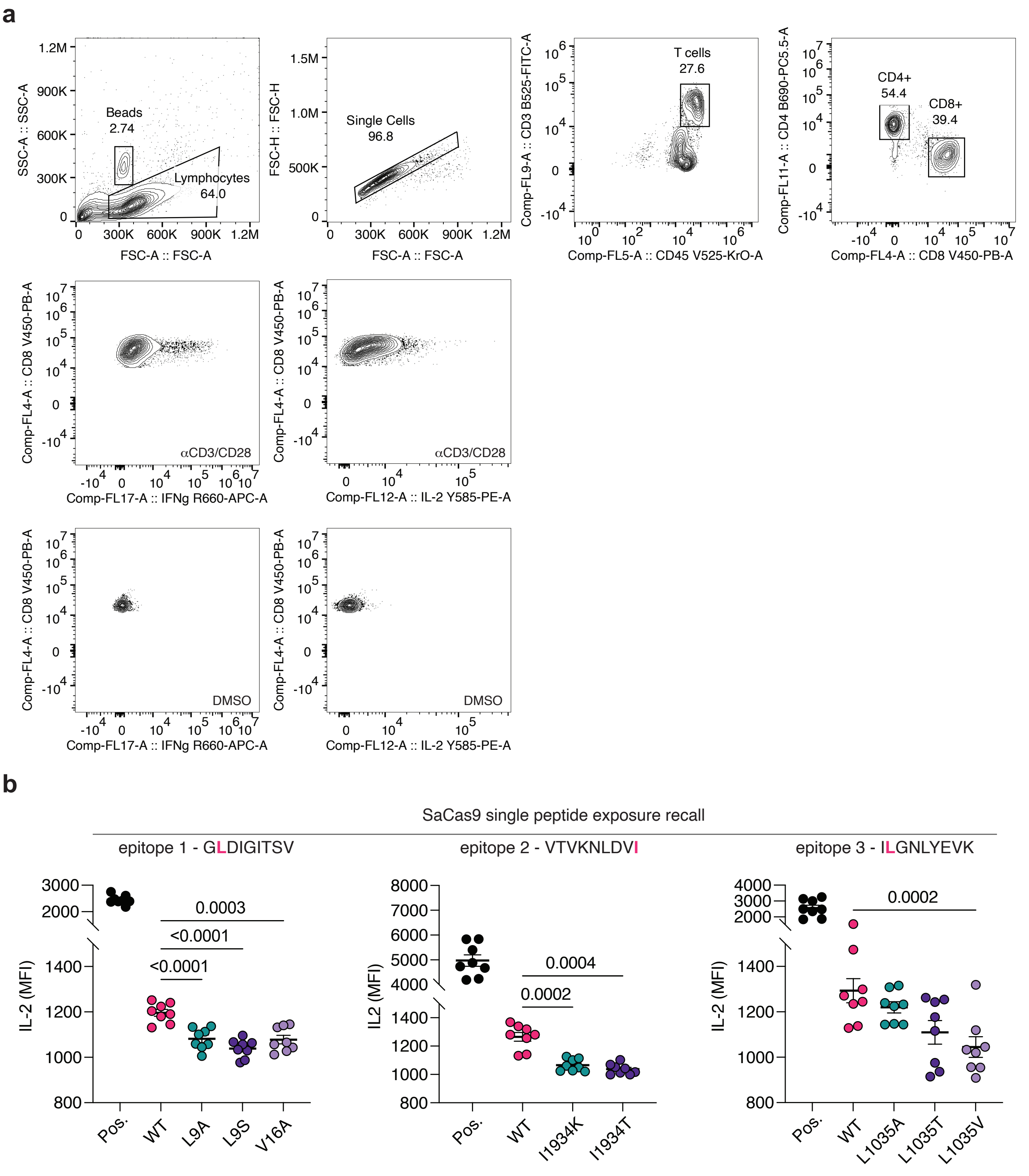

Supplementary Figure 7. Cytokine recall of SaCas9 memory responses in MHC-I/II-humanized mice. (a) Representative flow cytometry gating strategy to detect IFNγ and IL-2 secretion by overnight incubation of splenocytes from WT SaCas9-exposed MHC-I/II humanized mice with SaCas9 peptides. **(b)**T cell IL-2 recall against SaCas9 WT epitopes 1-3 and their respective variants. N=8 animals. Mean +/- SEM shown. Statistical significance was determined by repeated-measures one-way ANOVA followed by Dunnett's post hoc test for multiple hypothesis correction.

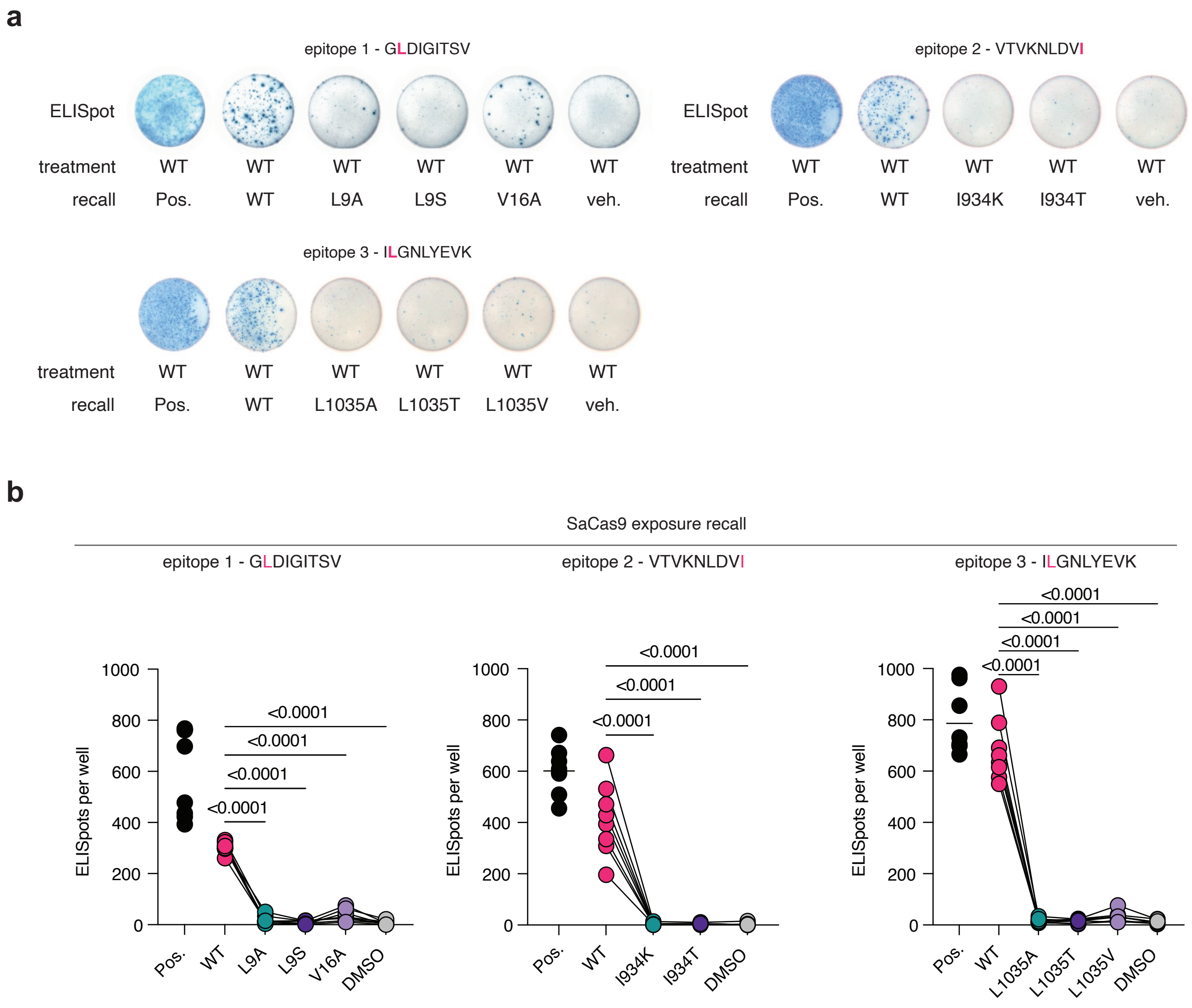

Supplementary Figure 8. ELISpot quantification with MHC-I/II-humanized and SaCas9-exposed splenocytes. (a) Representative ELISpot quantification for one animal immunized with WT SaCas9. Pos., anti-CD3/CD28 stimulation beads; Veh., vehicle (DMSO). **(b)** ELISpot recall against WT epitopes 1-3 and their respective variants. N=8 animals. Mean +/- SEM shown. Statistical significance was determined by repeated-measures one-way ANOVA followed by Dunnett's post hoc test for multiple hypothesis correction. Pos., anti-CD3/CD28 stimulation beads.

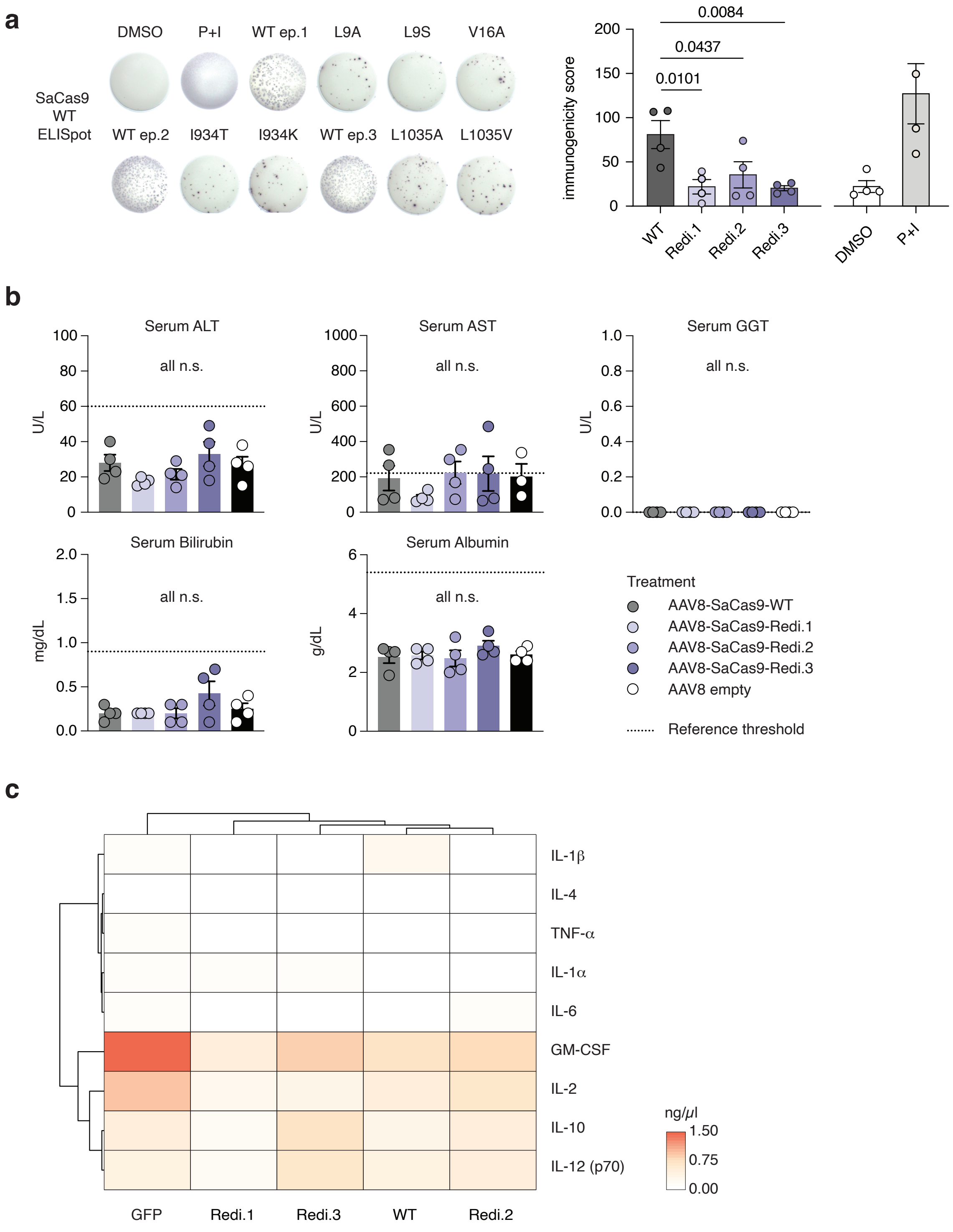

Supplementary Figure 9. Immunogenicity and liver toxicity profiles of AAV-delivered wild-type SaCas9 and SaCas9.Redi variants. (a) Representative IFN-γ ELISpot images for WT SaCas9 exposed splenocytes after T cell recall with immunodominant WT epitopes 1-3 and mutant peptides. **(b)** Bar charts indicating biochemical measurements of serum ALT, AST, albumin, and total bilirubin. Reference thresholds for adult C57BL/6J mice indicated as dotted lines. Sera from n=4 animals per condition were used as biological replicates. Statistical significance was determined by one-way ANOVA followed by Sidak post hoc test for multiple hypothesis correction. **(c)** Multiplex cytokine measurement in sera of AAV8-treated animals. Heatmap depicting median concentrations of indicated inflammatory cytokines measured in sera of animals treated with AAV-SaCas9-WT (n=3) and AAV-SaCas9.Redi1-3 (n=3 each).

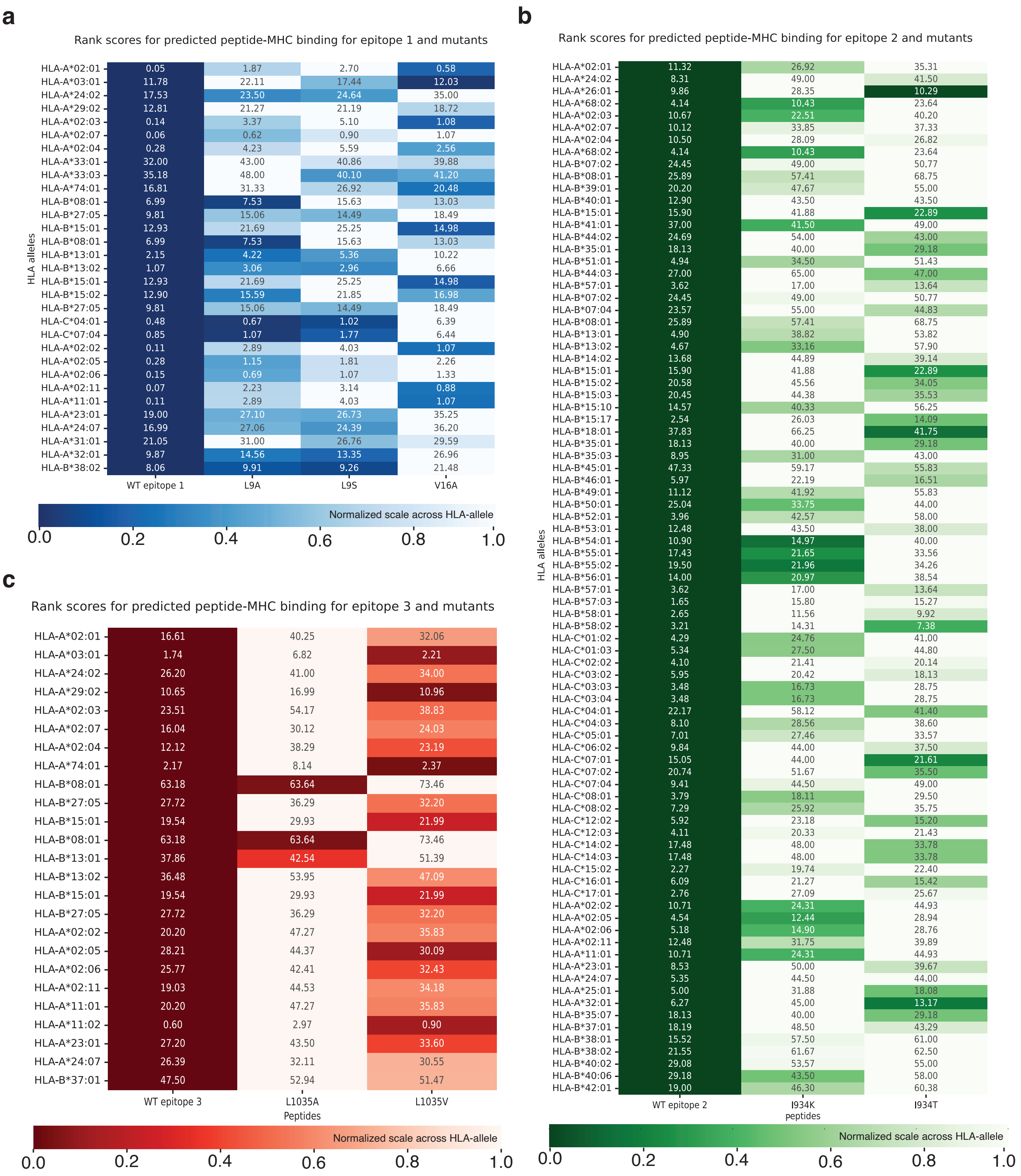

Supplementary Figure 10. NetMHCpan 4.1 predicted peptide-MHC binding for each epitope and Redi variants across relatively abundant HLA alleles. (a) Predicted peptide-MHC binding rank scores for WT epitope 1, and corresponding Redi.Cas9 mutant peptide sequences L9A, L9S and V16A. Heatmap coloring is normalized across each HLA-allele. **(b)** Predicted peptide-MHC binding rank scores for WT epitope 2, and corresponding Redi.Cas9 mutant peptide sequences I934K and I934T. Heatmap coloring is normalized across each HLA-allele. **(c)** Predicted peptide-MHC binding rank scores for WT epitope 3, and corresponding Redi.Cas9 mutant peptide sequences L1035A and L1035V. Heatmap coloring is normalized across each HLA-allele.

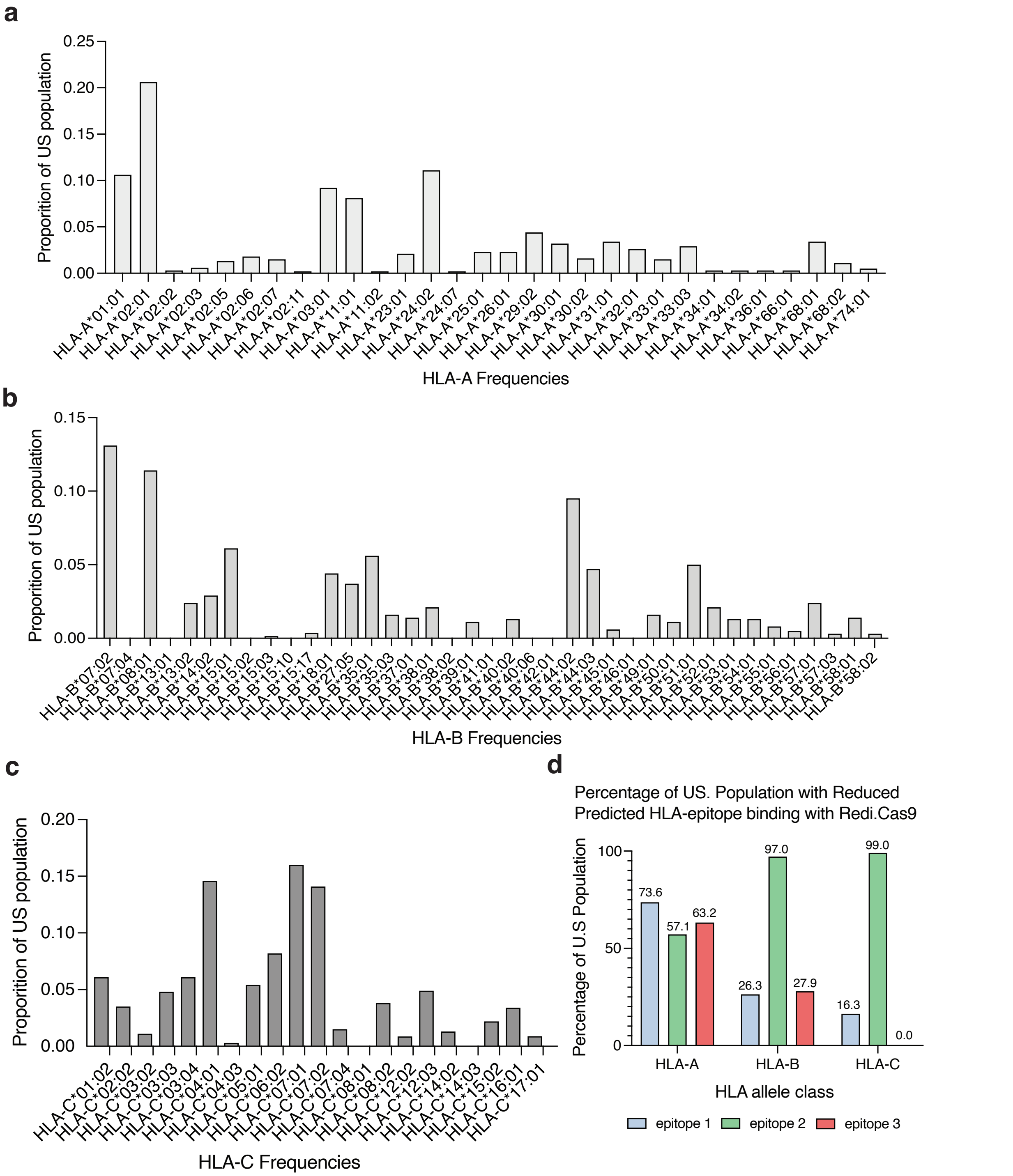

Supplementary Figure 11. Proportion of U.S population with predicted reduced HLA-peptide binding with Redi.Cas9 variants. (a) Proportion of the U.S population with the corresponding HLA-A class allele. Data derived from The Allele Frequency Net Database. **(b)** Proportion of the U.S population with the corresponding HLA-B class allele. Data derived from The Allele Frequency Net Database. **(c)** Proportion of the U.S population with the corresponding HLA-C class allele. Data derived from The Allele Frequency Net Database. **(d)** Percentage of U.S population with reduced predicted HLA-peptide binding with Redi.Cas9 variants as compared to WT epitopes as determined by relative increase in NetMHC4.1 rank score.

Supplementary Table 1. Computationally predicted binding affinities for SaCas9 and AsCas12a

SaCas9

peptide	seq	Mutation	Nat Binders	Mut Binders	Nat Strong Binders	Mut Strong Binders	Nat Global Binders	Mut Global Binders
8	GLDIGITSV	L9A	5	7	2	1	1599	1596
		L9F	5	6	2	0	1599	1601
		L9S	5	5	2	1	1599	1594
		V16A	5	4	2	1	1599	1597
		V16T	5	2	2	0	1599	1595
926	VTVKNLDVI	I934A	4	0	0	0	1599	1594
		I934S	4	0	0	0	1599	1593
		I934T	4	0	0	0	1599	1591
		I934K	4	2	0	0	1599	1595
1034	ILGNLYEVK	L1035A	1	0	0	0	1599	1597
		L1035T	1	1	0	0	1599	1597
		L1035V	1	1	0	0	1599	1597

AsCas12a

peptide	seq	Mutation	Nat Binders	Mut Binders	Nat Strong Binders	Mut Strong Binders	Nat Global Binders	Mut Global Binders
210	RLITAVPSL	L2111V,I212A	13	11	4	3	2231	2230
		L218T	13	4	4	1	2231	2224
		L211A,L218A	13	7	4	0	2231	2223
		L211V,L218A	13	9	4	0	2231	2227
		L218S	13	2	4	0	2231	2217
277	LNEVLNLAI	I285V	0	0	0	0	2231	2232
		I285T	0	0	0	0	2231	2230
		I285A	0	0	0	0	2231	2231
		I285S	0	0	0	0	2231	2230
972	YLSQVIHEI	L972F	10	9	3	3	2231	2233
		I979A	10	9	3	3	2231	2229
		I979T	10	3	3	1	2231	2221
		L972A,I979V	10	9	3	3	2231	2228
		I979A,I907F	10	9	3	3	2231	2223
		I979T,I907F	10	3	3	1	2231	2225
