# Supplementary material for: Rational engineering of minimally immunogenic nucleases for gene therapy
Source: Nat Commun. 2025 Jan 2;16:105. doi: 10.1038/s41467-024-55522-1 (PMC11696374; doi:10.1038/s41467-024-55522-1)
Supplement: Supplementary file 2 — Description of Additional Supplementary Files [file 41467_2024_55522_MOESM2_ESM.pdf]

Description of Additional Supplementary Files

File Name: Supplementary Data 1

Description: SaCas9 Mass Spectrometry Peptide Spectrum Match (PSM) results for replicate 1

File Name: Supplementary Data 2

Description: SaCas9 Mass Spectrometry Peptide Spectrum Match (PSM) results for replicate 2

File Name: Supplementary Data 3

Description: AsCas12a Mass Spectrometry Peptide Spectrum Match (PSM) results for replicate 1

File Name: Supplementary Data 4

Description: AsCas12a Mass Spectrometry Peptide Spectrum Match (PSM) results for replicate 2

File Name: Supplementary Data 5

Description: ELISpot quantification for SaCas9 and AsCas12a peptide treatment of PBMCs in vitro

File Name: Supplementary Data 6

Description: Indel rates of SaCas9 mutants

File Name: Supplementary Data 7

Description: Indel rates of AsCas12a mutants

File Name: Supplementary Data 8

Description: Predicted NetMHCpan rank scores across enriched HLA-alleles

File Name: Supplementary Code

Description: HLA-allele frequencies in the U.S Population
