# Supplementary material for: Rational engineering of minimally immunogenic nucleases for gene therapy
Source: Nat Commun. 2025 Jan 2;16:105. doi: 10.1038/s41467-024-55522-1 (PMC11696374; doi:10.1038/s41467-024-55522-1)
Supplement: Supplementary file 12 — Reporting Summary [file 41467_2024_55522_MOESM12_ESM.pdf]

nature portfolio

Corresponding author(s): Feng Zhang

Last updated by author(s): Oct 22, 2024

Reporting Summary

Nature Portfolio wishes to improve the reproducibility of the work that we publish. This form provides structure for consistency and transparency in reporting. For further information on Nature Portfolio policies, see our Editorial Policies and the Editorial Policy Checklist.

Statistics

For all statistical analyses, confirm that the following items are present in the figure legend, table legend, main text, or Methods section.

n/a	Confirmed	
☐	☒	The exact sample size (*n*) for each experimental group/condition, given as a discrete number and unit of measurement
☐	☒	A statement on whether measurements were taken from distinct samples or whether the same sample was measured repeatedly
☐	☒	The statistical test(s) used AND whether they are one- or two-sided<br>*Only common tests should be described solely by name; describe more complex techniques in the Methods section.*
☒	☐	A description of all covariates tested
☒	☐	A description of any assumptions or corrections, such as tests of normality and adjustment for multiple comparisons
☐	☒	A full description of the statistical parameters including central tendency (e.g. means) or other basic estimates (e.g. regression coefficient) AND variation (e.g. standard deviation) or associated estimates of uncertainty (e.g. confidence intervals)
☐	☒	For null hypothesis testing, the test statistic (e.g. *F*, *t*, *r*) with confidence intervals, effect sizes, degrees of freedom and *P* value noted<br>*Give P values as exact values whenever suitable.*
☐	☒	For Bayesian analysis, information on the choice of priors and Markov chain Monte Carlo settings
☒	☐	For hierarchical and complex designs, identification of the appropriate level for tests and full reporting of outcomes
☒	☐	Estimates of effect sizes (e.g. Cohen's *d*, Pearson's *r*), indicating how they were calculated

Our web collection on statistics for biologists contains articles on many of the points above.

Software and code

Policy information about availability of computer code

Data collection	Rosetta, NetMHCpan 4.1, ChimeraX - all publicly available
Data analysis	MATLAB, CRISSPRESSOv2, ImmunoSpot Analyzer, BELYSA® 1.1.0 software - all publicly available; custom Python script - included in the Methods

For manuscripts utilizing custom algorithms or software that are central to the research but not yet described in published literature, software must be made available to editors and reviewers. We strongly encourage code deposition in a community repository (e.g. GitHub). See the Nature Portfolio guidelines for submitting code & software for further information.

Data

Policy information about availability of data

All manuscripts must include a data availability statement. This statement should provide the following information, where applicable:

- Accession codes, unique identifiers, or web links for publicly available datasets
- A description of any restrictions on data availability
- For clinical datasets or third party data, please ensure that the statement adheres to our policy

All data supporting the findings of this study are available within the paper and its Supplementary Information. The mass spectrometry proteomics data have been deposited to the ProteomeXchange Consortium via the PRIDE partner repository with the dataset identifier PXD054579.

Human research participants

Policy information about studies involving human research participants and Sex and Gender in Research.

Reporting on sex and gender	Sex and gender are not reportedused in this study.
Population characteristics	HLA-typing for all patient samples are provided in the Supplementary information
Recruitment	Participants were not recruited through C.T.L
Ethics oversight	Ethical and regulatory compliance conducted by C.T.L

Note that full information on the approval of the study protocol must also be provided in the manuscript.

Field-specific reporting

Please select the one below that is the best fit for your research. If you are not sure, read the appropriate sections before making your selection.

☒ Life sciences ☐ Behavioural & social sciences ☐ Ecological, evolutionary & environmental sciences

For a reference copy of the document with all sections, see nature.com/documents/nr-reporting-summary-flat.pdf

Life sciences study design

All studies must disclose on these points even when the disclosure is negative.

Sample size	Using three technical replicates for indel sequencing and ELISpot analysis balances statistical rigor with resource efficiency while allowing detection of technical variability and assessment of data quality. This practice aligns with historical precedent and ethical considerations, ensuring reliable results while minimizing the number of experimental subjects. While larger sample sizes may offer more robust statistical analyses, three replicates are commonly accepted as the minimum necessary for meaningful interpretation in molecular biology and immunology assays.
Data exclusions	Wells with too many spots to reliably count using the ImmunoSpot Analyzer were excluded from analysis
Replication	All in vitro experiments were repeated multiple (>3) times to ensure that trends in nuclease efficacy were consistent. Reproducibility is confirmed by performing replicates of the sequencing reactions, validating results through comparison with control samples and non-targeting controls, and ensuring consistency in experimental conditions such as nuclease concentration and reaction conditions. For ELISpot assays, reproducibility is assessed by conducting technical replicates, validating results through comparison with known positive and negative controls, and ensuring consistency in assay conditions such as cell concentration. For in-vivo experiments, reproducibility is verified through randomization, blinding, and inclusion of appropriate controls to minimize bias and ensure consistency across experimental conditions. Groups sizes are reported in the manuscript, and the number of technical replicates is also indicated where appropriate.
Randomization	in vitro experiments were not randomized; for in vivo experiments, the animals were randomized to the different experimental conditions, with the investigator not blinded to the assignments
Blinding	in vitro experiments were not blinded; for in vivo experiments, the animals were randomized to the different experimental conditions, with the investigator not blinded to the assignments

Reporting for specific materials, systems and methods

We require information from authors about some types of materials, experimental systems and methods used in many studies. Here, indicate whether each material, system or method listed is relevant to your study. If you are not sure if a list item applies to your research, read the appropriate section before selecting a response.

Materials & experimental systems

n/a	Involved in the study	
☐	☒	Antibodies
☐	☒	Eukaryotic cell lines
☒	☐	Palaeontology and archaeology
☐	☒	Animals and other organisms
☒	☐	Clinical data
☒	☐	Dual use research of concern

Methods

n/a	Involved in the study	
☒	☐	ChIP-seq
☒	☐	Flow cytometry
☒	☐	MRI-based neuroimaging

Antibodies

Antibodies used

Anti-mouse CD4 PerCp clone: RM4-5 lot: 2279727, dilution: 1:200
Anti-mouse CD8a clone: eFluor 450 53-6.7 lot: 2527379, dilution: 1:100
Anti-mouse IL-2 PE clone: JES6-5H4 Rat IgG2b, k lot: B377599, dilution: 1:100
Anti-mouse CD45 BV510 clone: 30.F11 Rat IgG2b, k lot: B360620, dilution: 1:100
Anti-mouse IFN-γ APC clone: XMG-1.2 Rat IgG1, k lot: B370994, dilution: 1:100
Anti-mouse TNF-α PE/Cyanine7 clone: MP6-XT22 Rat IgG1, κ lot: B357460, dilution: 1:100
TruStain FCX (anti-mouse CD16/32) clone: 93 Rat IgG2a, λ lot: B372578, dilution: 1:50

Validation

PerCp Rat Anti-mouse CD4 has been validated internally with FACS-sorted positive T cell controls and by the manufacturer https://www.bdbiosciences.com/en-us/products/reagents/flow-cytometry-reagents/research-reagents/single-color-antibodies-ruo/percp-rat-anti-mouse-cd4.553052

Anti-Mo CD8a eFluor 450 has been validated internally with FACS-sorted positive T cell controls and by the manufacturer https://www.thermofisher.com/antibody/product/CD8a-Antibody-clone-53-6-7-Monoclonal/48-0081-82

Anti-mouse IL-2 PE has been validated internally using activated T cell controls in each experiment and by the manufacturer https://www.biolegend.com/en-us/products/pe-anti-mouse-il-2-antibody-954

Anti-mouse CD45 BV510 has been validated internally with FACS-sorted positive T cell controls and by the manufacturer https://www.biolegend.com/en-us/products/brilliant-violet-510-anti-mouse-cd45-antibody-7995

Anti-mouse IFN-γ APC has been validated internally using activated T cell controls in each experiment and by the manufacturer https://www.biolegend.com/en-us/products/apc-anti-mouse-ifn-gamma-antibody-993

Anti-mouse TNF-α PE/Cyanine7 has been validated internally using activated T cell controls in each experiment and by the https://www.biolegend.com/nl-be/products/pe-cyanine7-anti-mouse-tnf-alpha-antibody-5866

Eukaryotic cell lines

Policy information about cell lines and Sex and Gender in Research

Cell line source(s)

MDA-MB-231 - ATCC #HTB-26
HEK-293FT - Life Technologies R70007

Authentication

Authentication by comparing the STR profile of sample cell lines with the ATCC Human Cell STR Database

Mycoplasma contamination

all cell lines tested negative for mycoplasma contamination

Commonly misidentified lines
(See ICLAC register)

no commonly misidentified cell lines were used in the study

Animals and other research organisms

Policy information about studies involving animals; ARRIVE guidelines recommended for reporting animal research, and Sex and Gender in Research

Laboratory animals

HLA-A*0201 HLA-DRA*0101 HLA-DRB1*0101 transgenic mice devoid of mouse MHC [A2.DR1 mice, B6-Tg(HLA-DRA*0101, HLA-DRB1*0101)1Dmz Tg(HLA-A/H2-D/B2M)1Bpe H2-Ab1tm1Doi B2mtm1Unc H2-D1tm1Bpe] were provided by M. Berard and bred at the DKFZ animal facility 27. Mice were housed under Specific and Opportunistic Pathogen Free (SOPF) conditions and 12-hour day/night cycles

Wild animals

n/a

Reporting on sex

Sex was not considered in study design and mice of mixed sexes were accordingly ordered and used in the experiments described in this manuscript. Both female and male littermate mice were housed under Specific and Opportunistic Pathogen Free (SOPF) conditions and 12-hour day/night cycles. All experiments were conducted with sex-matched animals, without bias to either sex. Sex-based analysis was not performed.

Field-collected samples

n/a

Ethics oversight

AAV SaCas9 delivery experiments were approved by the governmental authorities (Regional Administrative Authority Karlsruhe, Germany) overseeing the German Cancer Research Center (DKFZ). SaCas9 peptide vaccination experiments were approved by the Institutional Animal Care and Use Committee (IACUC) of the Broad Institute (Protocol ID 0017-09-14-2). Animal maintenance complied with all relevant ethical regulations and were consistent with local, state and federal regulations as applicable, including the National Institutes of Health Guide for the Care and Use of Laboratory Animals.

Note that full information on the approval of the study protocol must also be provided in the manuscript.
